# An Antiquated Concept in the Novel Era of Ablation: Zero-fluoroscopy Pulsed Field Ablation for Treatment of Atrial Fibrillation

**DOI:** 10.19102/icrm.2025.16052

**Published:** 2025-05-15

**Authors:** Wissam Harmouch, Servando Cuellar, Arun Narayanan, Haider Al Taii, Muhie Dean Sabayon

**Affiliations:** 1 Department of Internal Medicine, University of Texas Medical Branch, Galveston, TX, USA; 2 Division of Cardiovascular Medicine, University of Texas Medical Branch, Galveston, TX, USA

**Keywords:** Ablation, atrial fibrillation, intracardiac echocardiography, pulsed field ablation, zero fluoroscopy

## Abstract

Intracardiac echocardiography (ICE) is a common tool that has real-time impact in novel pulsed-field ablation (PFA). It is a feasible and efficient option due to zero-fluoroscopy, real-time tissue visualization of procedural maneuvers, and for the assessment of potential procedural complications. We present a case of zero-fluoroscopy–based PFA using four-dimensional (4D) ICE in a 68-year-old man with symptomatic atrial fibrillation. Using 4D ICE, we were able to achieve procedural success by visualization of direct tissue contact with the Farawave™ catheter (Boston Scientific, Marlborough, MA, USA) with each rotation and application of the basket and flower configurations and no edema or color change in tissue morphology after applications. Overall, zero fluoroscopy in PFA is feasible and efficient.

## Introduction

Advancements in the field of electrophysiology (EP) have led to the use of pulsed field ablation (PFA) in the EP laboratory for the treatment of atrial fibrillation (AF). PFA exposes tissue to electrical gradients that cause tissue-specific irreversible electroporation in cardiac myocytes, which ultimately leads to apoptosis.^1^ Given the tissue specificity with PFA, damage to surrounding structures, such as the esophagus, phrenic nerve, and pulmonary veins, is less compared to that with other modalities, such as thermal ablation and cryoablation.^2^ The procedural time has been consistently lower with PFA compared to previous methods of ablation; however, the fluoroscopy time seems to be comparable to that of cryoablation and higher when compared to that seen with radiofrequency ablation.^3,4^ Given the known deleterious effects of radiation exposure, we present a case where we performed zero-fluoroscopy PFA using four-dimensional (4D) intracardiac echocardiography (ICE) to ensure safe and efficacious ablation in real time.

## Case presentation

A 68-year-old man with a remote history of paroxysmal AF with a CHA_2_DS_2_-VASc score of 1 point and premature ventricular complexes presented to our clinic with symptoms of palpitations. An electrocardiogram (ECG) at the time revealed normal sinus rhythm. To further investigate his symptoms and potential arrhythmias, the patient wore a 30-day monitor. This revealed underlying sinus bradycardia at 50–59 bpm with paroxysmal runs of AF ranging from 7–90 min with frequent episodes of rapid ventricular response ranging between 112 and 149 bpm. Additionally, echocardiography was obtained, which revealed an ejection fraction of 50%–55%, no significant valvular disease, and a left atrial (LA) volume index of 38.5 mL/m^2^. Given his frequent symptomatic AF and underlying sinus bradycardia, pulmonary vein isolation ablation was pursued.

The patient presented to the EP laboratory, where an ECG was recorded, which showed AF. The patient was administered anesthesia, prepped, and draped in a sterile manner. The right common femoral vein was accessed twice under ultrasound guidance, and two sheaths were placed. Perclose of these two access points was performed using Perclose ProGlide™ devices (Abbott, Chicago, IL, USA). One was deployed for each access point. Heparin was then administered, and an activated clotting time (ACT) of >300 s was maintained throughout the procedure. One milligram of atropine was then intravenously administered. This was performed to increase the patient’s heart rate throughout the case and to prevent significant vagal responses after PFA applications.

A 4D Nuvision ICE catheter (Biosense Webster, Diamond Bar, CA, USA) was then advanced into the right atrium (RA) and positioned to visualize the fossa ovalis. The ICE catheter confirmed that the LA appendage was free from thrombus. A Faradrive sheath with Faraconnect (Boston Scientific, Marlborough, MA, USA) was then advanced to the superior vena cava. A Baylis transseptal needle wire (Boston Scientific) was placed into this sheath and gradually positioned at the fossa ovalis under ICE guidance. Appropriate tenting of the fossa ovalis was then confirmed, and a transseptal puncture was performed **(Video 1)**. The Faradrive sheath was subsequently advanced into the LA to dilate the septum, then retracted back to the RA, and the Nuvision 4D ICE catheter was subsequently advanced into the LA. We advanced this catheter by visualizing, tracking, and following the Baylis transseptal wire under ICE. After positioning the ICE catheter in the body of the LA, the Faradrive sheath was then advanced into the LA over the Baylis transseptal wire. A Farawave™ PFA catheter (Boston Scientific) was then advanced into the Faradrive sheath.

**Video 1:** Tenting of Fossa Ovalis and Successful Transeptal Puncture. Appropriate tenting of the fossa ovalis was confirmed and a transeptal puncture was then performed and confirmed. Watch videos here: https://www.dropbox.com/scl/fo/bd5xegak417v1wwf91m0j/ACDCBL00gLNw-XZ3DUku0Yk?rlkey=0tospa7vowcx1if5qe548vuw7&e=1&st=5nw975hy&dl=0

The Rhythmia HDx™ mapping system (Boston Scientific) was used to construct a three-dimensional (3D) electroanatomic map of the LA using ICE and intracardiac electrograms (EGMs) that were recorded from the Farawave™ catheter. The mapping system was configured based on an impedance-based algorithm to visualize the catheter. The pulmonary veins were identified using ICE. A total of four veins were identified: right superior, right inferior, left superior, and left inferior pulmonary veins. All four pulmonary veins received a total of eight PFA applications each. Two applications were administered in the basket configuration, and two additional applications were administered after catheter rotation. The basket configuration was then changed into a flower configuration, where two applications were administered, and two additional applications were delivered after rotation of the catheter. Tissue contact was confirmed with each rotation of the catheter by visualization with ICE. This visualization was performed via two-dimensional (2D), 3D, and 4D ICE imaging as well as triplane imaging **(Video 2)**, which is a unique capability of the Nuvision ICE catheter. The 4D ICE mode allowed for the visualization of direct contact of the petals of the Farawave™ catheter in its flower configuration **(Video 3)**. This mode provided useful assessment of contact of the flower petals with the antrum of the veins. A 4D live visualization also allowed for the assessment of how coaxial the Farawave™ flower petals were to the antrum of the vein and whether one of the flower petals was inadvertently misplaced into the lumen of the vein rather than its antrum **(Video 4)**. After delivering all applications, no edema, morphologic changes, or acute procedural complications were visualized by color ICE.

**Video 2:** Intracardiac Echocardiography Confirming Tissue Contact via 2D, 3D, and 4D. Tissue contact was confirmed with each ration of the catheter by visualization with ICE. This visualization was performed via 2D, 3D and 4D ICE imaging as well as Triplane imaging. Watch videos here: https://www.dropbox.com/scl/fo/bd5xegak417v1wwf91m0j/ACDCBL00gLNw-XZ3DUku0Yk?rlkey=0tospa7vowcx1if5qe548vuw7&e=1&st=5nw975hy&dl=0

**Video 3:** Intracardiac Echocardiography Confirming Direct Tissue Contact in the Flower Configuration. The 4D ICE mode allowed for visualization of direct contact of the petals of the Farawave catheter in its flower configuration. Watch videos here: https://www.dropbox.com/scl/fo/bd5xegak417v1wwf91m0j/ACDCBL00gLNw-XZ3DUku0Yk?rlkey=0tospa7vowcx1if5qe548vuw7&e=1&st=5nw975hy&dl=0

**Video 4:** 4D Real-Time Visualization of the Farawave Flower Petals. The 4D visualization allowed for assessment of how coaxial the Farawave flower petals were to the antrum of the vein and whether one of the flower petals were inadvertently misplaced into the lumen of the vein rather than its antrum. Watch videos here: https://www.dropbox.com/scl/fo/bd5xegak417v1wwf91m0j/ACDCBL00gLNw-XZ3DUku0Yk?rlkey=0tospa7vowcx1if5qe548vuw7&e=1&st=5nw975hy&dl=0

After completion of the procedure, exit block was confirmed by pacing all four pulmonary veins via the Farawave™ catheter. All four pulmonary veins were isolated. The Faradrive sheath was then withdrawn from the LA and positioned in the inferior vena cava. The ICE catheter was then retracted into the RA. A final sweep of the pericardium was performed in the triplane mode, revealing no evidence of any pericardial effusion **(Video 5)**. A complete EP study with LA pacing was then performed with the atrial–His bundle interval at 105 ms and the His bundle–ventricular myocardium interval at 50 ms. No sustained tachycardia was able to be induced. After completion of the study, a repeat ACT was drawn, and protamine was given. After 10 min from protamine administration, the sheaths were removed, and Perclose sutures were deployed. Excellent hemostasis was achieved. The patient was transferred to recovery. A postoperative echocardiogram was performed prior to discharge, and there was no evidence of post-procedural pericardial effusion. The patient was then discharged home after 4 h and continued to do well post-procedure without complications. An ECG recorded post-procedure revealed sinus rhythm. One-month post-PFA, the patient continued to be in sinus rhythm, with no more episodes of palpitations, and with improved quality of life.

**Video 5:** Triplane Intracardiac Echocardiography Confirming No Pericardial Effusion. A final sweep of the pericardium was performed in Triplane mode which demonstrated no evidence of any pericardial effusion. Watch videos here: https://www.dropbox.com/scl/fo/bd5xegak417v1wwf91m0j/ACDCBL00gLNw-XZ3DUku0Yk?rlkey=0tospa7vowcx1if5qe548vuw7&e=1&st=5nw975hy&dl=0

## Discussion

Our case of zero fluoroscopy using 4D ICE with the Farapulse™ PFA system highlights a promising emerging modality for the treatment of AF. Furthermore, given the known harmful nature of radiation, our case is of paramount importance as it shows the feasibility of zero fluoroscopy in a common procedure via a novel modality, PFA. The concept of zero fluoroscopy in ablation procedures is not novel as previous groups, such as Hirata et al.^5^ and Ahn et al.,^6^ have studied the pragmatic nature of ICE-based ablations in AF. The procedural times and complications were not significantly different compared to fluoroscopy-based groups, but overall radiation exposure was much less.^6^ Zero fluoroscopy is not only vital concerning the “as low as reasonably achievable” concept but also regarding procedural efficiency.

Other tools, such as multi-spline catheters, including OCTARAY™ (J&J Medtech, New Brunswick, NJ, USA), ORION™ (Boston Scientific), and Advisor™ HD Grid mapping catheters (Abbott), can be beneficial in the evaluation of the elimination of fibrillatory circuits. These multi-spline catheters have been used successfully in PFA for patients with persistent AF.^7,8^ In addition to multi-spline catheters, mapping systems have become more advanced, allowing for accurate catheter visualization without the use of fluoroscopy. The Rhythmia navigation system allows for the visualization of the catheter tip by outlining the geometry of the atria via impedance measurements.^9^ The OPAL HDx™ Mapping System (Boston Scientific) is a newer mapping system that uses a similarly based algorithm to outline the geometry of the atria, but, compared to Rhythmia HDx™, it displays the exact shape of the Farawave™ catheter, whether in the flower-based configuration or basket-based configuration. Overall, multi-spine catheters and advanced mapping systems are potential tools in achieving zero fluoroscopy in PFA in patients with AF.

Furthermore, ICE has the capability to assist proceduralists in the visualization of endocardial structures, such as the fossa ovalis for transseptal punctures, LA, and ostia of pulmonary veins in real time.^10,11^ It can also assist in the assessment of post-procedural tissue morphology, by inspecting for edema, bubble formation, and potential complications like pulmonary vein stenosis and pericardial effusions.^11,12^ Utilizing 4D ICE allows for a real-time 3D assessment of cardiac function and procedural efficacy. This novel technology has been implemented in structural and EP cases. However, its implementation in PFA for AF is not well documented in the literature. Our case highlighted all these important concepts, as procedural success was achieved with the sole use of 4D ICE and EGMs. Real-time visualization of tissue contact with each configuration of the Farawave™ PFA catheter, each rotation, and application allowed for a faster procedural time compared to fluoroscopy-based PFA. Additionally, this technique in PFA allows for a direct assessment of potential acute procedural complications, by visualizing tissue morphology. We achieved this as we did not note any edema or color change to the tissue post-PFA. In the literature, one small case series in Europe showed similar findings as we have portrayed.^13^ Of note, in the Assessment of Safety and Effectiveness in Treatment Management of Atrial Fibrillation with the Biosense Webster Irreversible Electroporation Ablation System (AdmIRE) trial,^14^ a multicenter, single-arm study that aimed to evaluate the long-term safety and efficacy of PFA with integrated mapping, PFA was performed without fluoroscopy in approximately 25% of cases. However, discussion on the exact zero-fluoroscopy methods used was not explicitly stated. Our case highlights the clinical efficacy of zero fluoroscopy and bridges trial findings with clinical application. Overall, our case illustrates the feasibility and efficiency of zero-fluoroscopy PFA using 4D ICE for the treatment of AF.

## References

[1] Kotnik T, Rems L, Tarek M, Miklavčič D (2019). Membrane electroporation and electropermeabilization: mechanisms and models. Annu Rev Biophys.

[2] Verma A, Haines DE, Boersma LV (2023). PULSED AF Investigators. Pulsed field ablation for the treatment of atrial fibrillation: PULSED AF pivotal trial. Circulation.

[3] Urbanek L, Bordignon S, Schaack D (2023). Pulsed field versus cryoballoon pulmonary vein isolation for atrial fibrillation: efficacy, safety, and long-term follow-up in a 400-patient cohort. Circ Arrhythm Electrophysiol.

[4] Badertscher P, Weidlich S, Serban T (2023). Pulsed-field ablation versus single-catheter high-power short-duration radiofrequency ablation for atrial fibrillation: procedural characteristics, myocardial injury, and mid-term outcomes. Heart Rhythm.

[5] Hirata S, Nagashima K, Watanabe R (2024). Workflow of the zero-fluoro pulsed field ablation. J Arrhythm.

[6] Ahn J, Shin DG, Han SJ, Lim HE (2023). Safety and efficacy of intracardiac echocardiography-guided zero-fluoroscopic cryoballoon ablation for atrial fibrillation: a prospective randomized controlled trial. Europace.

[7] Magni FT, Scherr D, Manninger M (2023). Electrophysiological findings during re-do procedures after single-shot pulmonary vein isolation for atrial fibrillation with pulsed field ablation. J Interv Card Electrophysiol.

[8] Gunawardene MA, Schaeffer BN, Jularic M (2022). Pulsed-field ablation combined with ultrahigh-density mapping in patients undergoing catheter ablation for atrial fibrillation: practical and electrophysiological considerations. J Cardiovasc Electrophysiol.

[9] Cauti FM, Rossi P, Iaia L, Bianchi S (2020). A new mapping method with the Rhythmia™ navigation system reduces radiation exposure. Preliminary experience in SVT procedures. J Electrocardiol.

[10] Baykaner T, Quadros KK, Thosani A (2020). Safety and efficacy of zero fluoroscopy transseptal puncture with different approaches. Pacing Clin Electrophysiol.

[11] Dello Russo A, Russo E, Fassini G (2013). Role of intracardiac echocardiography in atrial fibrillation ablation. J Atr Fibrillation.

[12] Saliba W, Thomas J (2008). Intracardiac echocardiography during catheter ablation of atrial fibrillation. Europace.

[13] Rauber M, Manninger M, Eberl AS, Scherr D (2024). Zero-fluoroscopy ablation with multielectrode pulse field ablation system: case series. Pacing Clin Electrophysiol.

[14] Reddy VY, Calkins H, Mansour M (2024). Pulsed field ablation to treat paroxysmal atrial fibrillation: safety and effectiveness in the AdmIRE pivotal trial. Circulation.

